# WHO response to WTO member state challenges on tobacco, food and beverage policies

**DOI:** 10.2471/BLT.19.231985

**Published:** 2019-09-30

**Authors:** Pepita Barlow, Ronald Labonte, Martin McKee, David Stuckler

**Affiliations:** a Department of Health Policy, London School of Economics and Political Science, Houghton Street, Holborn, London WC2A 2AE, England.; b School of Epidemiology and Public Health, University of Ottawa, Ottawa, Canada.; c Department of Health Services Research and Policy, London School of Hygiene & Tropical Medicine, London, England.; d Carlo F Dondena Centre for Research on Social Dynamics and Public Policy, Bocconi University, Milan, Italy.

In 2013, the World Health Assembly endorsed the World Health Organization’s (WHO) *Global action plan for the prevention and control of noncommunicable diseases 2013–2020* to achieve a 25% reduction in mortality from noncommunicable diseases by 2025.^1^ Two years later, all of the world’s governments committed to reducing the global burden of noncommunicable diseases as part of the sustainable development goals. The rationale for these commitments is clear: in 2016, noncommunicable diseases caused almost three-quarters of all deaths worldwide and this burden has significant economic costs.^2^ The World Economic Forum estimates that, without concerted action, cumulative economic losses from noncommunicable diseases will exceed 7 trillion United States dollars over the period 2011–2025 in low- and middle-income countries.^3^

WHO’s Global Action Plan is ambitious, as it aims to achieve a world free of the avoidable burden of noncommunicable diseases.^1^ The plan recognizes that this aim can only be achieved through determined action by Member States and international partners. The contribution of WHO is its ability to convene, set norms and standards, and offer technical support. For example, WHO has developed cost–effective interventions for preventing noncommunicable diseases that include labelling regulations for salt, fat and sugar, for soft drinks, for tobacco and for alcoholic beverages.^3^

## Trade debates on tobacco policy

In the late 1990s, WHO used its treaty-making powers to address the issue of tobacco use, leading to the Framework Convention on Tobacco Control (FCTC).^4^ The FCTC has enabled WHO to have a greater presence at World Trade Organization (WTO) meetings, supporting countries in their efforts to protect their populations against the harms from tobacco. Governments might need this support when other members invoke WTO rules to challenge their public health policies based on their purported trade costs. Such challenges may arise even if the policies do not necessarily conflict with WTO requirements, for example if novel approaches are proposed, the application of the rules is uncertain or governments misrepresent WTO rules.^5^^,^^6^

We have reviewed the archives of tobacco and nutrition policy discussions at WTO’s Technical Barriers to Trade Committee, where WHO has an observer status through the WHO and Food and Agriculture Organization’s joint Codex Alimentarius Commission. This committee is the most likely to receive informal trade challenges to new tobacco, food and beverage policies proposed by WTO members. We identified 93 challenges that took place between 1995 and 2016 and found that the number of challenges per year increased from zero in 1995 to a high of 14 in 2014.^6^ Table 1 summarizes the challenges that were raised against regulations targeting these products, including infant milk formulae, alcoholic beverages, soft drinks, manufactured food products and their ingredients, cigarettes, tobacco, and cigarette flavourings.

**Table 1:** Regulated food, beverage and tobacco products that were subject to trade challenges at the World Trade Organization, 1995–2016

Product	Description	No. of challenges
Food	Food products, including processed foods, and their ingredients	46
Beverages	Alcoholic beverages, soft-drinks, fruit juices and other non-alcoholic beverages, infant milk formulae	36
Tobacco	Tobacco, tobacco flavourings, cigarettes	15

Note: The number of trade challenges on each product category exceeds the total number of challenges (93) because some challenges were about regulations that affected several products.

Source: Barlow et al. 2018.^6^

At least 15 debates about policies affecting trade in tobacco products were raised at WTO meetings, although only one escalated to a formal dispute under WTO dispute settlement rules (and a WTO panel eventually ruled in favour of the policy). WHO has attended WTO debates about tobacco and defended, challenged or disputed tobacco control measures by citing the FCTC. For example, in November 2012 the Dominican Republic, Honduras, Nicaragua, Nigeria and others challenged New Zealand’s proposal to introduce plain packaging of tobacco products. These countries cited the financial impact of the measure on low- and middle-income countries, and questioned the scientific basis of the proposal.^7^ WHO was present at the committee meeting and commented on New Zealand’s proposal and challenges, citing evidence about harms from tobacco and the effectiveness of such legislation.^7^ WHO further noted that the packaging proposal would be consistent with New Zealand’s requirement to fulfil its obligations under the FCTC, which requires parties to adopt effective measures with labelling and packaging, and implement comprehensive bans on tobacco promotion. When similar proposals to introduce plain packaging of tobacco products were presented by France (2014), Norway (2015) and Singapore (2016), WHO again made formal submissions, presenting similar evidence and citing relevant provisions in the FCTC.^8^

## Trade debates on nutrition policy

While WHO was present when tobacco trade may conflict with public health concerns, this was not the case in WTO discussions concerning nutrition policy. Our analysis showed that between 1995 and 2016 there were 82 challenges to regulations affecting food and beverage products at the Technical Barriers to Trade Committee.^9^ Some health measures challenged included those in WHO’s list of cost–effective interventions for preventing noncommunicable diseases. Forty-seven (57%) of these challenges were against labelling regulations, with 24 (29%) on quality standards and restrictions on certain products or ingredients.

These policies are portrayed in the challenges as non-tariff barriers to trade, with countries arguing that they create unnecessary costs; that the evidence is weak or inadequate; or that there could be a less trade-restrictive alternative. Another 11 (13%) challenges questioned the regulation’s rationale or legitimacy. Major power imbalances exist within the committee: high-income countries raised most trade challenges against health measures proposed or in place in low- and lower-middle-income countries. An example is Indonesia’s 2013 proposal to introduce warning labels to describe high levels of sugar, fat and salt content in processed foods. Australia, Canada and the European Union challenged the measure, questioning its scientific justification, although food labelling is now known to be effective in encouraging consumers to choose healthier products.^10^ These countries requested that Indonesia consider an alternative measure such as an education campaign.^11^

None of these challenges to food and beverage policies ultimately escalated to a formal trade dispute, overseen by a panel of tribunal trade specialists. However, a challenge can be enough to make a country back down, especially in low- and middle-income countries and when the challenge is from an economically and politically powerful country. We are concerned that governments could acquiesce to such pressures, simply because of the economic and political costs of resisting, and because the outcome of a formal trade dispute is uncertain. We were not able to identify the outcome of every challenge because outcomes are not systematically recorded. However, our archival research identified at least five cases where pressure at WTO was followed by a delay, change or abandonment of the initial policy.^6^

## Increasing WHO participation

Even though the *Global action plan for the prevention and control of noncommunicable diseases 2013–2020,* fully recognizes the need for action on trade in certain foods and beverages, we were unable to find any evidence of WHO participation in nutrition-related trade challenges, such as those related to unhealthy food high in salt, fat and sugar, alcohol, soft-drinks and infant milk formulae. WHO can learn from its past successes in championing tobacco control at the WTO. WHO has a responsibility to refute false claims, especially those that challenge the adequacy of evidence supporting particular policies, such as alcohol beverage labelling regulations, marketing and labelling requirements for energy drinks and regulations for front-of-pack nutrition labelling for food products. For example, some governments have argued that daily meal guides and education campaigns are effective in preventing obesity. Yet there is extensive evidence that these approaches have very little, if any, impact.^12^

There may be several reasons for WHO’s limited engagement in nutrition policy debates. First, WHO does not have formal or ad hoc observer status in the committee, limiting scope for its participation independent of the Codex Alimentarius. WHO should be able to participate in meetings, to counter arguments against effective dietary or food-related health measures, whenever these are being challenged at WTO. WHO could also request full observer status at the committee, giving it a voice on matters of direct interest to WHO’s goals.^9^ Obtaining an observer status would allow WHO to inform the committee’s debates. 

Second, WHO may not be fully aware of the extent to which the committee’s discussions focus on relevant policies. Third, WHO may face human resource and capacity (expertise) constraints to participation. Fourth, Member States may not always agree on how to respond to challenges concerning nutrition-related policies. This lack of consensus may happen because they disagree or are uncertain about how much sugar, salt or fat concentration is detrimental and how much is acceptable, or because relevant national or international guidelines have not yet been developed.

WHO faces other challenges in extending its ability to defend tobacco control measures to nutrition regulations. The lack of a treaty similar to the FCTC for nutrition-related diseases may discourage WHO participation because such absence limits the perceived legitimacy of WHO input. The political economy of tobacco production and control also differs from food and beverage production and policy. Most challenges to tobacco policies were raised by low- and middle-income countries with large tobacco sectors against high-income countries, whereas many challenges to nutrition policies were raised by high-income countries against low- and middle-income countries. High-income countries may have been more effective in requesting support from WHO in defending their tobacco policies. In addition, tobacco control concerns the producers of one commodity, whereas nutrition policy affects numerous products sold by transnational food corporations with complex supply chains.

Further investigations are necessary to understand why WHO has yet to comment on food and beverage regulations at WTO’s committee. We nevertheless believe that WHO should have the opportunity to attend and speak at the committee’s debates, if efforts to combat noncommunicable diseases are to succeed. 
